# A Feasibility Study on Deep Learning Based Brain Tumor Segmentation Using 2D Ellipse Box Areas

**DOI:** 10.3390/s22145292

**Published:** 2022-07-15

**Authors:** Muhaddisa Barat Ali, Xiaohan Bai, Irene Yu-Hua Gu, Mitchel S. Berger, Asgeir Store Jakola

**Affiliations:** 1 Department of Electrical Engineering, Chalmers University of Technology, 41296 Gothenburg, Sweden; barat@chalmers.se (M.B.A.); hadleybai0913@gmail.com (X.B.); 2 Department of Neurological Surgery, University of California San Fransisco, San Francisco, CA 94143-0112, USA; mitchel.berger@ucsf.edu; 3 Department of Clinical Neuroscience, University of Gothenburg, 40530 Gothenburg, Sweden; jakola.asgeir@gu.se; 4 Department of Neurosurgery, Sahlgrenska University Hospital, 41345 Gothenberg, Sweden

**Keywords:** 2D ellipse box areas, multi-stream U-Net, brain tumors, glioma segmentation, MR images, deep learning

## Abstract

In most deep learning-based brain tumor segmentation methods, training the deep network requires annotated tumor areas. However, accurate tumor annotation puts high demands on medical personnel. The aim of this study is to train a deep network for segmentation by using ellipse box areas surrounding the tumors. In the proposed method, the deep network is trained by using a large number of unannotated tumor images with foreground (FG) and background (BG) ellipse box areas surrounding the tumor and background, and a small number of patients (<20) with annotated tumors. The training is conducted by initial training on two ellipse boxes on unannotated MRIs, followed by refined training on a small number of annotated MRIs. We use a multi-stream U-Net for conducting our experiments, which is an extension of the conventional U-Net. This enables the use of complementary information from multi-modality (e.g., T1, T1ce, T2, and FLAIR) MRIs. To test the feasibility of the proposed approach, experiments and evaluation were conducted on two datasets for glioma segmentation. Segmentation performance on the test sets is then compared with those used on the same network but trained entirely by annotated MRIs. Our experiments show that the proposed method has obtained good tumor segmentation results on the test sets, wherein the dice score on tumor areas is (0.8407, 0.9104), and segmentation accuracy on tumor areas is (83.88%, 88.47%) for the MICCAI BraTS’17 and US datasets, respectively. Comparing the segmented results by using the network trained by all annotated tumors, the drop in the segmentation performance from the proposed approach is (0.0594, 0.0159) in the dice score, and (8.78%, 2.61%) in segmented tumor accuracy for MICCAI and US test sets, which is relatively small. Our case studies have demonstrated that training the network for segmentation by using ellipse box areas in place of all annotated tumors is feasible, and can be considered as an alternative, which is a trade-off between saving medical experts’ time annotating tumors and a small drop in segmentation performance.

## 1. Introduction

Brain tumor segmentation from MR images (MRIs) is an important step toward clinical assessment, determining treatment strategies, and performing further tumor tissue analysis. Many automatic methods have been successfully used for tumor segmentation. However, most of these methods need tumor data annotations by medical experts, which is a time-consuming process. Apart from this, these methods are also prone to intra- and inter-observer variability [1,2]. Recently, deep learning methods have drawn much attention for tumor segmentation when a large training dataset is available. Among these methods, the first used U-Net [3], and its variants [4,5] were most frequently reported due to their good performance on medical image segmentation. Wang et al. [6] proposed brain-wise normalization and two patching strategies for training a 3D U-Net. Kim et al. [7] introduced a two-step setup for the segmentation task, wherein an initial segmentation map was obtained from 2D U-Nets which together with the MRIs are further used by 3D U-Net for the final segmentation map. Shi et al. [8] used an increased number of channels in its proposed U-Net, which is capable of extracting rich and diverse features from multi-modality scans. Other deep learning methods such as CNNs [9,10,11] were also shown to be useful. For example, Sun et al. [12] proposed a computationally efficient custom-designed CNN with a reduced number of parameters. Das et al. [13] used 3D CNN in a cascaded format to extract whole tumors first in a series followed by the core tumor and then the enhanced core tumor. Shan et al. [14] proposed a lightweight 3D CNN with improved depth and used multi-channel convolution kernels of different sizes to aggregate features. Ramin et al. [15] used a cascade CNN to speed up the learning. However, these deep learning approaches often require all annotated tumors for training the network, and manually annotating tumors for training datasets is a time-consuming process.

There exist many successful studies on non-medical images in computer vision where information has been acquired from unannotated images e.g., bounding boxes [16,17,18], and image-level and point-level labeling [19,20,21], among many others. Rectangular bounding boxes were used for object detection and tracking based on the Riemannian manifold learning of dynamic visual objects [22,23]. However, in medical applications, such approaches are still being exploited. Zhang et al. [24] proposed a semi-supervised method that exploits information from unlabeled data by estimating segmentation uncertainty in predictions, and Luo et al. [25] used a dual-task deep network to predict a segmentation map and geometry-aware level set labels. Ali et al. proposed the use of rectangular shape [26] and ellipse shape [27] bounding box tumor regions for tumor classification. Pavlov et al. [28] used ResNet50 for segmentation with both tumor ground truth and image-level annotation. Zhu et al. [29] developed a segmentation method that was guided by image-level class labels on 3D cryo-ET images. Xu et al. [30] suggested a method called “3D-BoxSup” by using 3D bounding box labels for MRI brain tumor segmentation, with relatively low performance (dice score = 0.62 on MICCAI’17 dataset). This was probably due to the fact that 3D models required more training data and also the fact that the pure use of bounding boxes was not sufficient to obtain an irregular tumor shape estimation. It is worth noting that although the use of bounding box areas for training machine learning/deep learning networks is widely used for object tracking from visual images in computer vision, it is rarely used for medical MR image segmentation. Some reasons could be that MR images are very different from visual images and also the lack of medical experts’ knowledge, which causes the gap between the medical research and computer vision communities.

Motivated by the above issues, we propose the performance of tumor segmentation, whereby we train the deep network by using tumor ellipse box areas instead of MRIs with annotated tumors. The main aims of this study are 1) to investigate whether the paradigm of brain tumor segmentation, based primarily on using large numbers of ellipse box areas for tumors in MR images, plus a small number of annotated tumor patients, is feasible, and 2) to answer the question of what price one needs to pay when replacing the annotated MRIs for training the network in brain tumor segmentation. Because U-Net has demonstrated excellent performance for medical image segmentation, a multi-stream U-Net (an extension of U-Net) is employed in our case studies, wherein combined features from multiple MRI modalities will be explored. The main contributions of this paper are as follows.

- We study the feasibility of the use of 2D ellipse box areas for training the deep network for brain tumor (glioma) segmentation plus a small number of annotated tumors.
- We use a multi-stream U-Net for our experiments, which is an extended version of the conventional U-Net.
- We conduct studies on two scenarios: (a) if the training dataset is large/moderate, learning is conducted by pre-training on a large amount of FG and BG ellipse areas followed by refined-training on a small number of annotated tumor patients (<20); and (b) if the training dataset is small, learning is conducted in a fashion similar to the idea of transfer learning.
- We evaluate the performance of the proposed approach and compare the performance with the same network trained entirely by using annotated MRIs.

The remainder of the paper is organized as follows. In Section 2, the proposed method is described in detail, including the framework for case studies, the FG–BG ellipse area definition, the multi-stream U-Net, training strategies for large/medium and small datasets, and several other issues. Section 3 gives experimental results, performance evaluation, and comparison, and is followed by Section 4, with a conclusion.

## 2. Proposed Method

**Figure 1:**
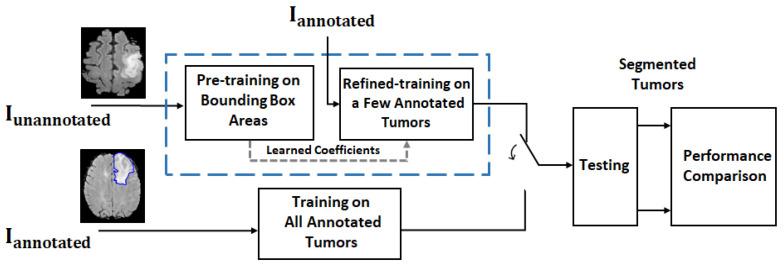
Framework for a feasibility study on MR brain tumor segmentation. For the proposed deep learning approach (blue dash line box), the training process consists of 2 rounds: coarse training on unannotated MRIs $I_{unannotated}$ with FG-BG ellipse box areas and refined training on a few annotated MRIs $I_{annotated}$. The trained network is then used for tumor segmentation on the test dataset. For performance comparison, the deep network with the same structure trained on all annotated MRIs $I_{annotated}$ is also implemented (see the block under the blue dash line box) for comparison purposes, as it provides the best test results (i.e., segmentation results) under the same structured deep network. The segmentation results are then compared.

The proposed approach is based on the hypothesis that it is feasible to train a deep network for brain tumor (glioma) segmentation by training a large percentage of unannotated brain tumor MRIs $I_{unannotated}$ by using ellipse box areas surrounding the tumors and background, and a small number of medical expert-annotated tumor patient MRIs $I_{annotated}$ (<20). The motivation is that if one can replace this ellipse box-based learning paradigm with acceptable tumor segmentation performance on the test set, then one would be able to save a lot of time from manual tumor annotation. In order to carry out such a study, a framework is depicted in Figure 1.

### 2.1. Defining Foreground and Background Ellipse Box Areas Using Ellipses

**Figure 2:**
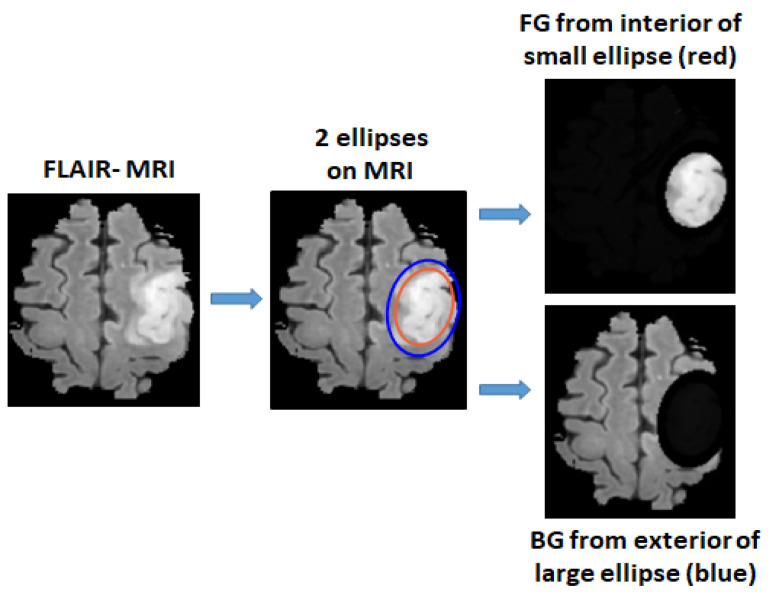
Foreground area (FG) and background area (BG) areas are defined by two ellipses, where FG is extracted from the interior area of a small ellipse surrounding the tumor and BG is extracted from exterior area of a larger ellipse.

For unannotated MRIs, the foreground (FG) and background (BG) areas are used as the inputs for the training. The FG area in a MRI is defined as the interior area of a small ellipse surrounding the tumor, and the BG area is defined as the exterior area of a large ellipse containing normal tissues, as shown in Figure 2. Pixels from the interior area of the small ellipse have a high probability of being the tumor pixels and are used for the initial training of positive tumor class, whereas the pixels from the exterior area of the large ellipse have a high probability of being the normal brain tissues and are used for the initial training of the negative non-tumor class. For the small ellipse, first an initial ellipse is drawn on the area surrounding the tumor. To minimize the non-tumor pixels, this ellipse is then shrunk by a scale factor $p_{1}$ (0.9 was used based empirical tests). The large ellipse is drawn with the same center as the initial ellipse, the axes of which are multiplied by a scale factor $p_{2}$ (1.2 was used based on empirical tests). In this way, the exterior area can avoid most tumor pixels. An ellipse is first drawn manually by selecting areas surrounding the tumor and then a Matlab functio, regionprops is used for estimating two ellipse axes for drawing 2 ellipses.

### 2.2. Multi-Stream U-Net Used for Our Experiments

**Figure 3:**
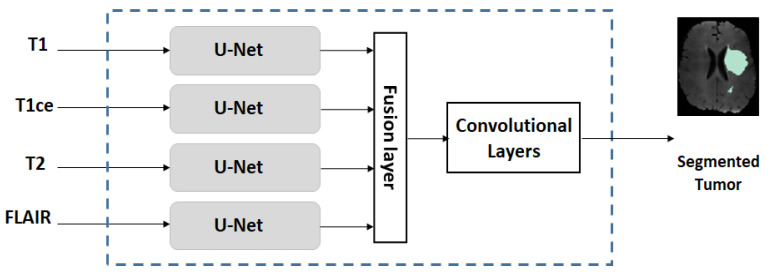
Structure of a multi-stream U-Net network.

Because different MRI modalities provide complementary information on tumors and because U-Net [3] is very successful for MR image segmentation, we decided to employ an extended U-Net, called multi-stream U-Net, for our case studies. A multi-stream U-Net contains several number of parallel U-Net (where the number is equal to the MRI modalities, e.g., a four-stream U-Net is used when T1, T1ce, T2, and FLAIR are available). This is followed by a simple feature-level fusion that combines the features from different modalities. Figure 3 shows the block diagram of the multi-stream U-Net. For the MICCAI dataset, the number of MRI modalities is four (where T1, T1ce, T2, and FLAIR are available) and for the US dataset the number of modalities used is two (where T1ce and FLAIR are available). Each single-stream U-Net has a symmetric structure consisting of downstream paths and upstream paths. Details of the architecture are summarized in Table 1.

### 2.3. Training Strategies on Datasets with Different Sizes

#### 2.3.1. Training on Dataset with Large/Moderate Size

When the dataset size is large or is of moderate size, the multi-stream U-Net is initially pre-trained on the large percentage (90%) of the training set with ellipse-defined foreground (FG) and background (BG) areas. Then, it is refined-trained on a small percentage (about 10%, or <20 patients) of the annotated training set.

#### 2.3.2. Training on a Small Dataset

Because using a small dataset is not sufficient to give a good training result in deep networks, we adopted an idea similar to “transfer learning” for training the small dataset. This is done by using the weights of the network training on a large/moderate size dataset (e.g., MICCAI dataset) as the initial weights, followed by applying the refined training on the given specific small dataset (which updates the weights on all network layers). We note that this is different from the conventional transfer learning approach, where only the weights on a few top layers would be updated. The reason for this difference is due to a domain mismatch issue when several datasets are combined. Because most datasets were captured from somewhat different domains (e.g., from different institutions with different scanner parameter settings), simply merging them to a enlarge the data in order to obtain improved test results would not work well. Domain adaptation is usually required before merging several training datasets [26]. This is reflected in our training method on updating weights in all layers, as weights in low layers could be more related to large changes due to different measurement domains, updating all weights would make the network better tuned to this specific given dataset. When the dataset is very small, we use a small number of patients (<20) whose tumors are annotated by radiologists for refined training. After refined training on the small dataset, network weights which are better tuned to the specific features in the small dataset are then fixed and used for the segmentation.

### 2.4. Other Issues

#### 2.4.1. Strict Patient-Separated Splitting of Training/Validation/Testing Sets

For a given training dataset, a strict patient-separated approach is applied when splitting the dataset into training, validation, and testing subsets. If the size of a dataset is large/moderate, we perform a dataset split to approximately (training, validation, and testing) = (60%, 20%, and 20%). This is to ensure that each patient’s data only occurs either in the training or in the testing, but not in both. If the size of dataset is very small, we simply split the dataset according to patients into training and testing categories equal to 20% and 80%, respectively, where tumor annotations are assigned to the training set and the remaining to the testing subset.

#### 2.4.2. Criteria for Performance Evaluation

Criteria used for evaluating the performance of tumor segmentation are given as follows.

##### Tumor Accuracy

This is the accuracy of the tumor area, which is the region of interest for tumor segmentation and is defined as follows:

$$\mathrm{Tumor}\;\mathrm{Accuracy}=\frac{\mathrm{TP}}{\mathrm{TP}\;+\;\mathrm{FN}},$$

where TP and FN denote the true positive (i.e., tumor pixels) and false negative, respectively.

##### Tumor Dice Score and Jaccard Index

The dice score is applied only on the tumor pixel areas to evaluate the tumor segmentation performance. Let *X* and *Y* be an annotated tumor image and the corresponding tumor segmented image, the dice score on tumor areas is defined as

$$D=\frac{2|X⋂Y|}{\left|X|+|Y\right|}.$$

The Jaccard similarity index is computed to find the similarity between *X* and *Y* and is given as

$$J=\frac{\left|X⋂Y\right|}{\left|X\cup Y\right|}.$$

## 3. Results and Performance Evaluation

### 3.1. Datasets, Setup, Pre-Processing

#### 3.1.1. Datasets

Experiments were conducted on two datasets: MICCAI BraTS’17 and US. The MICCAI dataset is an open dataset with a moderate number of patients consisting of four modalities (T1, T1ce, T2, FLAIR) MRIs on low-grade glioma (LGG) and high-grade glioma (HGG) [31,32,33]. The US dataset is a clinical, private dataset obtained from a US hospital, consisting of two modalities (T1ce, FLAIR) on LGG. For the US dataset, tumor boundaries around the whole tumor areas were marked manually by radiologists. Table 2 describes the detailed information on these two datasets. For testing the concept on the proposed approach, we merged the pixels from different sub-regions of a glioma such as the necrotic and non-enhancing pixels, the peritumoral edema and the enhancing pixels as the tumor pixels in the MICCAI dataset. This can mitigate the problem of imbalanced sub-classes in training by limiting the segmentation to just 2 classes (i.e., tumor/non-tumor) in both the datasets.

We used 2D slices instead of 3D scans as the input of the network in order to mitigate the possible overfitting in deep learning (if the dataset size is moderate/small) and to reduce the computation cost. For each 3D scan in the MICCAI dataset (moderate size), nine image slices are extracted from three views with a distance of five slices from both sides when keeping the one with the largest tumor area as the center slice, whereas for the US dataset (small size), 18 image slices are extracted from three views because the dataset is small. For all MRI scans in the MICCAI dataset, 60% (or 171 patients) were used for training which consists of 154 unannotated and 17 annotated patients. For the US dataset, 15 annotated patients were used for training, and the remaining 60 patients were used for testing.

#### 3.1.2. Setup

The Keras library on a backend TensorFlow is used on a GPU platform by using NVIDIA GeForce RTX 2080 Ti from Google Colab. It had a video RAM of 11GB with CPU 6× Xeon E5-2678 v3 and 62 GB memory. The network hyperparameters were empirically determined and chosen from the best-trained network. For the network parameters, in the multi-stream U-Net, the learning rate was set to 1.0 × 10${}^{-3}$. Adagrad was used as the optimizer. The batch size was set to 16. L2-norm regularization was applied with the value of the parameter selected as 1.0 × 10${}^{-3}$ for convolutional layers in each stream. The dropout rate was set as 10% at the end of the downstream path as described in [3]. Categorical cross-entropy was used as the loss function in the network. For the training process, 70 epochs were used for the first round of training, and 150 epochs for the second round of training. To balance the training samples in tumor/non-tumor areas, weighting factors were applied to FG and BG pixels. The weights were determined empirically based on the approximated ratio of the average number of FG and BG pixels. In addition, simple augmented images were added through horizontal and vertical flipping, shearing with $0.2^{\circ }$ rotation and scaling by a factor up to 10% during the training through Keras function ImageDataGenerator.

#### 3.1.3. Pre-Processing

Because MRI scans from the US dataset were not registered, pre-processing was performed on these 3D scans. This pre-processing included registration of anatomical images (from FLAIR and T1ce scans) to a 1-mm MNI template. Furthermore, bias field correction and skull-stripping were performed by using software packages [34,35]. No pre-processing was performed on the MICCAI dataset because they were already skull-stripped and co-registered to their T1-modality. Further, all 2D image slices were normalized in size (176 × 176) pixels, with zero mean and unit variance.

### 3.2. Results, Comparison and Discussion

#### 3.2.1. Results

The proposed paradigm was evaluated on the MICCAI and US datasets. For the MICCAI dataset, a four-stream network was trained first on MRIs from 154 patients without annotations, followed by refined training on annotated tumor MRIs from 17 patients. For the US dataset, we used a two-stream network trained on the MICCAI dataset (only on T1ce and FLAIR MRIs) as the initial network, followed by a refined training on annotated tumor MRIs from the US dataset to learn this dataset’s specific features. The performance on segmented tumor images on the two test sets (averaged on five runs, each time on a new patient-wise data subset followed by testing on the completely trained network) have shown good results. The average accuracy results are further split according to each class (i.e., tumor and non-tumor) and are described by the confusion matrix in Table 3.

A set of evaluation results, tumor accuracy and dice score are further included on the test sets of two datasets in Table 4.

Observing Table 4a, one can see that the averaged test accuracy on the positive tumor pixels (i.e., the region of interest) is 83.88% and 88.47% on the MICCAI and US dataset, respectively. In addition, one can see that the average dice scores computed on tumor areas are good (0.8407 and 0.9104), and the Jaccard index values on tumor areas are reasonably good (0.7233 and 0.8355) on the MICCAI and US test sets. Furthermore, Table 4b shows the sensitivity, specificity, and false positive rate from the confusion matrix in Table 3. Based on these evaluation results, the proposed method seems to have resulted in good tumor segmentation on both datasets.

**Figure 4:**
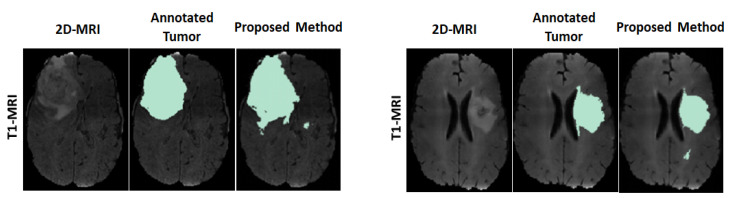
Example of two segmented brain tumor images from the MICCAI test set. Columns (on left and right): original T1 MR image; annotated tumor area marked by medical experts; segmented tumor area from proposed method.

For visual observation on the segmented tumors, Figure 4 shows an example of two segmented tumor images (column 3 on left and right) by using the proposed approach.

#### 3.2.2. Comparison

We then compare the performance of the proposed paradigm to that of the network trained on all annotated data, where it has the exact same network architecture as the multi-stream U-Net used in the case studies for the proposed paradigm. The only difference is that all training samples were obtained from the annotated tumor images (i.e., 100% of MRIs in the training dataset were annotated) in the latter case. The point of this comparison is to examine, by using the same network architecture, how much the performance degrades if the greater part of the training data is not annotated and to see if it is feasible to use such a paradigm. Table 5 shows the comparison of the test results in terms of tumor accuracy and tumor dice score on the MICCAI and US test datasets.

Observing Table 5, one can see that although the proposed approach has achieved good segmentation results, there is a slight performance degradation as compared with the results from the conventional method (i.e., network trained on MRIs where all tumors contain GT annotations). The degradation on average test results obtained are shown in bold fonts as 8.78 ± 0.07%, 0.0594 ± 0.0012 for the MICCAI test set and 2.61 ± 0.31%, 0.0159 ± 0.0003 for the US test set. It is rather encouraging to see the very small changes in the dice score, as the dice score is usually considered an important performance measure. The comparison indicates that the proposed method is rather effective based on these two datasets.

To further evaluate the proposed scheme, Table 6 shows the comparison of the dice scores from several state-of-the-art methods, as well as the method using fully annotated GT tumor areas for training (i.e., the “conventional” method). It is worth noting that the results from the methods [8,24] in Table 6 can only be used as an indication of performance because they were trained on a much larger BraTS’19 as comparing to the one using BraTS’17 [30]. Observing the results in bold fonts in Table 6, the “conventional” method resulted with the best segmented performance as 0.9001 and the proposed method as 0.8407.

#### 3.2.3. Discussion

In our case studies, experiments were conducted on two MRI datasets to check the feasibility of the proposed deep network learning approach for brain tumor segmentation. The aim is to see whether the proposed approach is feasible when the greater part of the training data is without GT tumor annotations. The proposed training method has led to a small performance drop as compared to that which uses a fully annotated tumor trained network. Our case studies have demonstrated that the proposed approach is feasible (though more extensive studies are needed on more datasets), and can be used as a tradeoff when tumor annotations on a large training dataset becomes a bottleneck. Further, a comparison with state-of-the-art methods shows its effectiveness.

## 4. Conclusions

Many medical datasets often lack annotated tumors because tumor annotation is a time-consuming process for medical experts. We conducted a feasibility study on two datasets (with glioma tumor type) by using ellipse box tumor areas for the initial training on majority training data followed by refined training by using annotated tumor MRIs from a small number of patients (<20). Experiments have shown good tumor segmentation results evaluated purely on tumor areas in terms of dice score (0.8407, 0.9104) and average accuracy (83.88%, 88.47%) for the MICCAI and US datasets, respectively, which demonstrated that the proposed approach is feasible by using a large amount of unannotated MRI data. Compared with the same network trained exclusively with annotated data, the proposed approach shows a small decrease in performance (a decrease in dice score = (0.0594, 0.0159) and a decrease in accuracy = (8.78%, 2.61%) for the MICCAI and US test sets). The proposed method provides an alternative approach, which is a tradeoff between a small decrease in performance, and saving time and manual labor for medical doctors. Future work will be conducted on more datasets.

## Figures and Tables

**Table 1:** Detailed architecture of a single stream U-Net used in the multi-stream U-Net.

Block No.	No. of Units
Downstream-path	Conv2D, stride, BN, Max-pooling
1	[3 × 3, 32, ReLU] × 2, 2, BN, 2 × 2
2	[3 × 3, 64, ReLU] × 2, 2, BN, 2 × 2
3	[3 × 3, 128, ReLU] × 2, 2, BN, 2 × 2
4	[3 × 3, 256, ReLU] × 2, 2, BN, 2 × 2
5	[3 × 3, 512, ReLU] × 2, 2, BN, -
Upstream-path	Conv2DTranspose, concat, BN, Conv2D
6	[2 × 2, 128], concat, -, [3 × 3, 256, ReLU] × 2
7	[2 × 2, 128], concat, BN, [3 × 3, 128, ReLU] × 2
8	[2 × 2, 128], concat, BN, [3 × 3, 64, ReLU] × 2
9	[2 × 2, 128], concat, BN, [3 × 3, 32, ReLU] × 2

**Table 2:** Summary of two datasets, as well as the number of 2D slices in each 3D scan, and information on patient-separated split of training/validation/testing subsets.

Dataset	T1/T1ce/T2/FLAIR	#2D	#2D */3D	#2D */3D	#2D */3D
		Slices	(Training)	(Validation)	(Testing)
MICCAI	285/285/285/285	9	1539/171	513/57	513/57
US	0/75/0/75	18	270/15	-	1080/60

* Excluded augmented 2D slice images

**Table 3:** Confusion matrices from the test results, by splitting the average tumor accuracy according to tumor and non-tumor areas. All results were averaged on five runs.

**(a) MICCAI dataset**		
Predicted\True	Tumor (±σ)	Non-tumor (±σ)
Tumor	83.88 (±0.08)	1.54 (±0.09)
Non-tumor	16.12 (±0.08)	98.46 (±0.09)
**(b) US dataset**		
Predicted\True	Tumor (±σ)	Non-tumor (±σ)
Tumor	88.47 (±0.34)	0.42 (±0.27)
Non-tumor	11.53 (±0.34)	99.58 (±0.27)

**Table 4:** Performance evaluation on the test set from using the proposed approach (averaged over five runs).

(**a**)			
Dataset	Tumor Accuracy (±σ)%	Dice Score (±σ)	Jaccard Index (±σ)
MICCAI	83.88 (±0.08)	0.8407 (±0.0006)	0.7233 (±0.0028)
US	88.47 (±0.34)	0.9104 (±0.0021)	0.8355 (±0.0029)
(**b**)			
Dataset	Sensitivity (±σ)%	Specificity (±σ)%	False Positive (±σ)%
MICCAI	83.88 (±0.08)	98.46(±0.09)	1.54 (±0.09)
US	88.47 (±0.34)	99.58 (±0.27)	0.42 (±0.27)

**Table 5:** Comparison of the test results averaged over five runs (accuracy and dice score) on the MICCAI and US dataset by using the proposed method and the conventional method (i.e., same deep network trained by all tumors with annotations). The degradation shows the performance difference on each dataset.

Datasets	Method	Tumor Accuracy % (±σ)	Dice Score (±σ)
MICCAI	Proposed	83.88 (±0.08)	0.8407 (±0.0006)
	Conventional	92.66 (±0.15)	0.9001 (±0.0018)
	Degradation	−8.78(±0.07)	−0.0594(±0.0012)
US	Proposed	88.47 (±0.34)	0.9104 (±0.0021)
	Conventional	91.08 (±0.21)	0.9263 (±0.0024)
	Degradation	−2.61(±0.31)	−0.0159(±0.0003)

**Table 6:** Comparison with existing state-of-the-art methods on the MICCAI BraTS dataset.

Method	Dataset	Dice Score
3D-BoxSup [30]	BraTS’17	0.6200
Semi-supervised [24]	BraTS’19	0.8361
Supervised [8]	BraTS’19	0.8645
Conventional (GT supervised training)	BraTS’17	**0.9001**
Proposed	BraTS’17	**0.8407**

## Data Availability

Datasets used in the paper was downloaded from BraTS brain tumor segmentation challenge (http://braintumorsegmentation.org/, accessed on 15 December 2021). Requests to access the datasets should be directed to them. US dataset is a private clinical dataset from a US hospital.

## Abbreviations

The following abbreviations are used in this manuscript:

BG	Background
BN	Batch normalization
CNN	Convolutional Netural Network
FG	Foreground
FLAIR	Fluid-Attenuated Inversion Recovery
FN	False negative
GT	Ground truth
HGG	High Grade Glioma
LGG	Low Grade Glioma
MICCAI	Medical Image Computing Computer Assisted Intervention Society
MNI	Montreal Neurological Institute
MRI	Magentic Resonance Image
ReLU	Rectifier Linear Unit
T1	T1 weighted
T2	T2 weighted
T1ce	T1 weighted with Contrast Enhanced
